# Caspase-3 and caspase-8 expression in breast cancer: caspase-3 is associated with survival

**DOI:** 10.1007/s10495-016-1323-5

**Published:** 2016-10-31

**Authors:** Xuan Pu, Sarah J. Storr, Yimin Zhang, Emad A. Rakha, Andrew R. Green, Ian O. Ellis, Stewart G. Martin

**Affiliations:** 1 Division of Cancer and Stem Cells, Department of Clinical Oncology, School of Medicine, Nottingham University Hospitals NHS Trust, City Hospital Campus, Nottingham, UK; 2 Department of Breast and Thyroid Surgery, Renmin Hospital, Wuhan University, Wuhan, Hubei China; 3 Division of Cancer and Stem Cells, Department of Histopathology, School of Medicine, University of Nottingham, Nottingham University Hospitals NHS Trust, City Hospital Campus, Nottingham, UK

**Keywords:** Breast cancer, Caspase, Calpain, Breast cancer-specific survival, Biomarker

## Abstract

Impaired apoptosis is one of the hallmarks of cancer. Caspase-3 and -8 are key regulators of the apoptotic response and have been shown to interact with the calpain family, a group of cysteine proteases, during tumorigenesis. The current study sought to investigate the prognostic potential of caspase-3 and -8 in breast cancer, as well as the prognostic value of combinatorial caspase and calpain expression. A large cohort (n = 1902) of early stage invasive breast cancer patients was used to explore the expression of caspase-3 and -8. Protein expression was examined using standard immunohistochemistry on tissue microarrays. High caspase-3 expression, but not caspase-8, is significantly associated with adverse breast cancer-specific survival (*P* = 0.008 and *P* = 0.056, respectively). Multivariate analysis showed that caspase-3 remained an independent factor when confounding factors were included (hazard ratio (HR) 1.347, 95% confidence interval (CI) 1.086–1.670; *P* = 0.007). The analyses in individual subgroups demonstrated the significance of caspase-3 expression in clinical outcomes in receptor positive (ER, PR or HER2) subgroups (*P* = 0.001) and in non-basal like subgroup *(P* = 0.029). Calpain expression had been previously assessed. Significant association was also found between high caspase-3/high calpain-1 and breast cancer-specific survival in the total patient cohort (*P* = 0.005) and basal-like subgroup (*P* = 0.034), as indicated by Kaplan–Meier analysis. Caspase-3 expression is associated with adverse breast cancer-specific survival in breast cancer patients, and provides additional prognostic values in distinct phenotypes. Combinatorial caspase and calpain expression can predict worse prognosis, especially in basal-like phenotypes. The findings warrant further validation studies in independent multi-centre patient cohorts.

## Introduction

Dysregulated apoptosis is a hallmark of human cancers. Two major pathways initiate apoptosis: the extrinsic [or death receptor (DR)] pathway is activated in response to ligand binding of DRs superfamily members, resulting in activation of caspase-8 followed by caspase-3; the intrinsic (or mitochondrial) pathway is triggered by mitochondrial release of cytochrome c, leading to formation of Apaf-1 and cytochrome c complex with the assistance of ATP, subsequently activating caspase-9 followed by caspase-3 (reviewed in [1]). Caspases are cysteine aspartyl proteases and 14 family members have been identified [2]. Based on their function, location and structural characteristics, caspases are generally classified as apoptotic caspases or pro-inflammatory caspases. Typical apoptotic caspases can be subdivided into two groups: the initiator caspases (i.e. caspase-2, -8, -9 and -10); and the effector caspases (i.e. caspase-3, -6 and -7). All caspases exist as inactive zymogens (procaspases) and their activation requires proteolytic activity during apoptosis. An effector caspase is activated by an initiator caspase through cleavage at the internal Asp residue, leading to disassembly of the large and small subunit; the inhibitor caspase, however, is activated by dimerization via the signal obtained from death receptors (reviewed in [3]).

Aberrant caspase expression and/or activation have been implicated in various types of cancer. High caspase-3 expression has been demonstrated in acute myelogenous leukaemia (AML) compared to the normal counterparts [4]. In comparison, reduced caspase-3 expression has been observed in moderately and poorly differentiated prostate cancer specimens compared with well-differentiated prostate tumours and normal tissues [5]. Loss of caspase-8 mRNA/protein expression has been demonstrated in high grade small cell lung cancers, neuroendocrine lung cancers and paediatric neuroblastoma. Previous studies have suggested that caspase-8 might act as a tumour suppressor in certain types of lung cancer and neuroblastoma [6, 7]. Studies have also shown that *CASP8* gene polymorphism may contribute to the increased risk of oesophageal squamous cell carcinoma [8, 9]. In breast cancer, in vitro studies have shown that dysregulated caspase activity is involved in chemotherapeutic resistance. One study demonstrated that restoration of caspase-3 expression, in caspase-3 deficient MCF-7 breast cancer cells, can sensitise to doxorubicin- and etoposide-induced apoptosis, suggesting caspase-3 deficiency may be a possible mechanism for chemoresistance [10, 11]. Furthermore, restoration of expression in MCF-7 cells restored cytochrome c- and caspase-8 -mediated activation of pro-caspase-9 [12].

The calpain family, a group of proteolytic intracellular cysteine proteases (EC 3.4.22.17 Clan CA, family C02), are calcium-activated and expressed in a wide range of cells and tissues [13]. Calpastatin is the only known endogenous inhibitor of calpain (reviewed in [14]). The calpain system is involved in the apoptotic machinery through interaction with caspase family members; and a number of caspase family members can be proteolytically processed by calpains. Inhibition of calpain in various tumour cell lines results in p53-dependent apoptosis, cell cycle arrest, and caspase activation (i.e. caspases-2, -3, -6, -8, and -9) [15]. Caspase-7 and -10 can be activated by calpain cleavage [16], calpain-mediated cleavage of caspase-12 is required for endoplasmic reticulum (ER) stress-induced apoptosis [17] and degradation of caspase-9 by calpain results in an inactivated form of the enzyme, unable to activate caspase-3 [18].

Previous studies have demonstrated that high caspase-3 expression is significantly associated with improved prognosis in patients with non-small cell lung cancer and hepatocellular carcinomas [19, 20]. The expression of caspase-3 and -6 in breast cancer was not associated with clinical outcome in a small study (n = 210) [21]. Calpain-1, -2 and calpastatin are extensively expressed in breast tumours, ovarian tumours, gastro-oesophageal tumours, pancreas, bile duct and ampulla tumours, and are associated with clinical outcome or treatment response [22–27]. The aim of the current study was to assess caspase-3 and -8 protein expression, their prognostic potential in early invasive breast cancer; as well as the importance of combinatorial caspase/calpain protein expression.

## Materials and methods

### Clinical samples

This immunohistochemical based study was performed using a cohort of 1902 early stage breast cancer patients treated at Nottingham University Hospitals between 1986 and 1998 with long term follow-up. Information on clinical history and outcome was maintained on a prospective basis, and patients’ clinical history and tumour characteristics were assessed in a standardised manner, including age at diagnosis, tumour size, histologic stage and grade, Nottingham prognostic index (NPI), lymphovascular invasion (LVI), oestrogen receptor (ER), progesterone receptor (PR) and human epidermal growth factor receptor 2 (HER2) status. The median age of the patients was 55 years (ranging from 18 to 72) and the median follow-up time was 177 months (ranging from 1 to 308 months). 63.2% (1203 of 1902) of patients had stage I disease. Patients were managed under a uniform protocol, where all underwent mastectomy (n = 1067, 56.1%) or wide local excision (n = 819, 43.1%) and approximately half of the patients received radiotherapy (n = 1025, 53.9%). Systemic adjuvant treatment was given dependent upon NPI values, ER and menopausal status. Patients with an NPI value <3.4 did not receive adjuvant chemotherapy, whereas patients with an NPI value of 3.4 or above were chosen for CMF chemotherapy (cyclophosphamide, methotrexate and 5-fluorouracil, n = 320, 16.8%) if they were ER negative or premenopausal; patients with ER positive disease were candidates for hormone therapy (n = 674, 35.4%).

Breast cancer-specific survival was defined as the time interval (in months) from the start of primary surgery to death resultant from breast cancer. ER, PR and HER2 status were available for this cohort and have been described previously [28]. HER2 expression was determined by immunohistochemistry with fluorescence in situ hybridisation (FISH) used as the arbiter in cases with an immunohistochemistry score of 2. Basal like phenotype was defined as the detection of cytokeratin (CK)-5/6 and/or CK-14 expression in 10% or more of invasive breast tumour cells, irrespective of ER, PR or HER2 status [29]. This study is reported in accordance with REMARK criteria [30]. Nottingham Research Ethics Committee 2 approved the project under “Development of a molecular genetic classification of breast cancer R&D (No. 03HI01 REC Ref.C202313)”. The clinicopathologic variables of the cohort are shown in Table 1.

**Table 1:** Clinicopathologic variables of patient cohort

Variables	No. (%)	Variables	No. (%)
Age (mean ± SD, years)	54.25 (±9.77)	ER status	
≤40	165 (8.7%)	Positive	1370 (72.0%)
>40	1736 (91.3%)	Negative	476 (25.0%)
ND	1 (0.1%)	ND	57 (3.0%)
Tumour size (mm)	2.06 ± 1.14	PR status	
≤20	1185 (62.3%)	Positive	1035 (54.4%)
>20	708 (37.2%)	Negative	739 (38.9%)
ND	9 (0.5%)	ND	128 (6.7%)
Tumour stage		HER2 status	
I	1203 (63.2%)	Positive	243 (12.8%)
II	531 (27.9%)	Negative	1602 (84.2%)
III	160 (8.4%)	ND	57 (3.0%)
ND	8 (0.4%)	Basal status	
Tumour grade		Positive	368 (19.3%)
I	346 (18.2%)	Negative	1390 (73.1%)
II	632 (33.2%)	ND	144 (7.6%)
III	915 (48.1%)	Triple negative status	
ND	9 (0.5%)	Positive	315 (16.6%)
NPI	4.16 ± 1.18	Negative	1516 (79.7%)
≤3.4	619 (32.5%)	ND	71 (3.7%)
3.41–5.4	948 (49.8%)	Breast cancer-specific survival	
>5.4	324 (17.0%)	Alive	1064 (55.9%)
ND	11 (0.6%)	Dead	505 (26.6%)
Lymphovascular invasion		ND	333 (17.5%)
Positive	492 (25.9%)	Recurrence	
Negative	1070 (56.3%)	Present	752 (39.5%)
ND	340 (17.9%)	Not present	1103 (58.0%)
Operation type		ND	47 (2.5%)
Mastectomy	1067 (56.1%)	Distant metastasis	
WLE lumpectomy	819 (43.1%)	Present	579 (30.4%)
ND	16 (0.8%)	Not present	1310 (68.9%)

Continuous data are shown as mean ± standard deviation (SD). *NPI* Nottingham prognostic value, *WLE* wide local excision, *ER* oestrogen receptor, *PR* progesterone receptor, *HER2* human epidermal growth factor receptor 2, *ND* not determined

### TMA construction and Immunohistochemistry

**Fig. 1:**
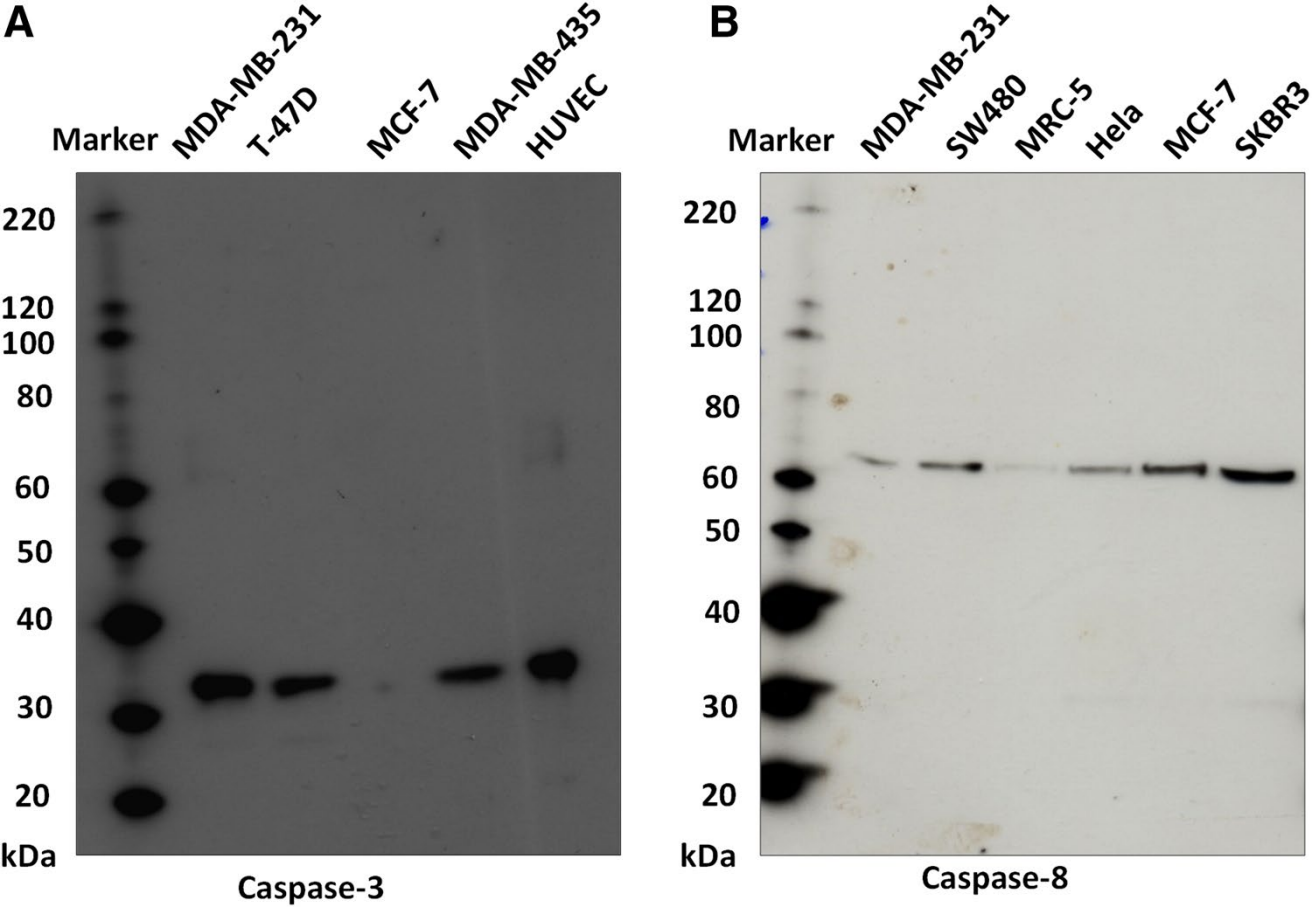
Antibody validation for anti-caspase-3 (*panel A*) and anti-caspase-8 (*panel B*). A single specific band, of 35 kDa, was obtained for caspase-3, and a single specific band, of 62 kDa, obtained for caspase-8. Expression, as shown, was assessed across a range of breast cancer cell lines, representing different phenotypes (MDA-MB-231, MDA-MB-435, T-47D, MCF-7 and SKBR3), human umbilical vein endothelial cells (HUVEC), fibroblasts (MRC-5), cervical cancer (HeLa) and/or colorectal cancer cells (SW480’s). Expression of caspase-3 was absent, as expected, from MCF-7’s

Caspase-3 and -8 protein expression was investigated using tissue microarrays (TMAs) by immunohistochemistry. All calpain and calpastatin protein expression was assessed previously [23, 31]. A single 0.6 mm tissue core was used for each patient with the core being taken from a representative tumour area as assessed by a specialist breast cancer histopathologist, as described previously [32]. Freshly cut 4 μm sections of the TMAs were used for immunohistochemistry. Primary antibody specificity was confirmed using Western blotting, on a range of breast cancer cell lines, prior to immunohistochemistry. Validation of anti-caspase-3 and -8 antibody specificity was conducted by Western blotting and data is shown in Fig. 1. Equal protein loading was used however without a loading control no firm conclusions can be drawn regarding the relative expression of the proteins across the different cell lines. Validation of anti-calpain-1, -2, -9 and calpastatin antibody specificities were established in previously published studies [22, 23, 31]. It should be noted that the anti-caspase-3 and -8 antibodies used in the current study can detect both full-length and cleaved forms of the respective proteins. Briefly, following dewaxing in xylene and rehydration in ethanol, antigen retrieval was performed in 0.01 mol/L sodium citrate buffer (pH 6.0) in a microwave, 750 W for 10 min followed by 450 W for 10 min. Caspase-3 and -8 staining were achieved using a Vectastain Universal Elite ABC Kit (Vector Laboratories, USA) and a Novolink Polymer Detection System (Leica, Denmark) following the manufacturers’ protocols, respectively. Caspase-3 (1:100, Cell Signalling Technology, USA, 9662) was applied to the tissue for 1 h at room temperature, and caspase-8 (1:25, Thermo Scientific, USA, PA1-29159) was applied to the tissue for overnight at 4 °C. Immunohistochemical reactions were visualised with 3,3′-diaminobenzidine and counterstained with haematoxylin, dehydrated and fixed in xylene followed by mounting with DPX. Positive controls constituted early stage breast tumour composite sections of varying grade and negative control omitted primary antibody.

Staining was assessed at 200× magnification following high-resolution scanning (Nanozoomer Digital Pathology Scanner, Hamamatsu Photonics). Protein expression was assessed using a semi-quantitative immunohistochemistry H-score as previously described [33]. The staining intensity was assessed as: none (0), weak (1), medium (2) and strong (3), and H-scores were calculated by multiplying the percentage of positive areas by the staining intensity, giving rise to a score ranging between 0 and 300. 30% of scores were examined by a second independent assessor, blind to clinicopathological and survival endpoints. Good concordance was demonstrated between both scorers (single measure intraclass correlation coefficients were 0.898 for caspase-3 and 0.732 for caspase-8). The cut point used to dichotomise immunohistochemical scores was determined in a non-biased fashion using X-tile software [34].

### Statistical analysis

The relationship between categorised protein expression and clinicopathologic factors were examined using Pearson’s Chi square test of association (χ^2^) or Fisher’s exact test if a cell count was <5 in a 2 × 2 table. Spearman’s rank test was performed to assess for correlations between the different protein expression levels. Survival curves were plotted using the Kaplan–Meier method and significance determined using the Log-rank test. Multivariate survival analysis used the Cox proportional hazards regression model. All differences were considered statistically significant at the level of *P* < 0.05. Statistical analysis was performed using SPSS 22.0 software.

## Results

### Immunohistochemical staining

**Fig. 2:**
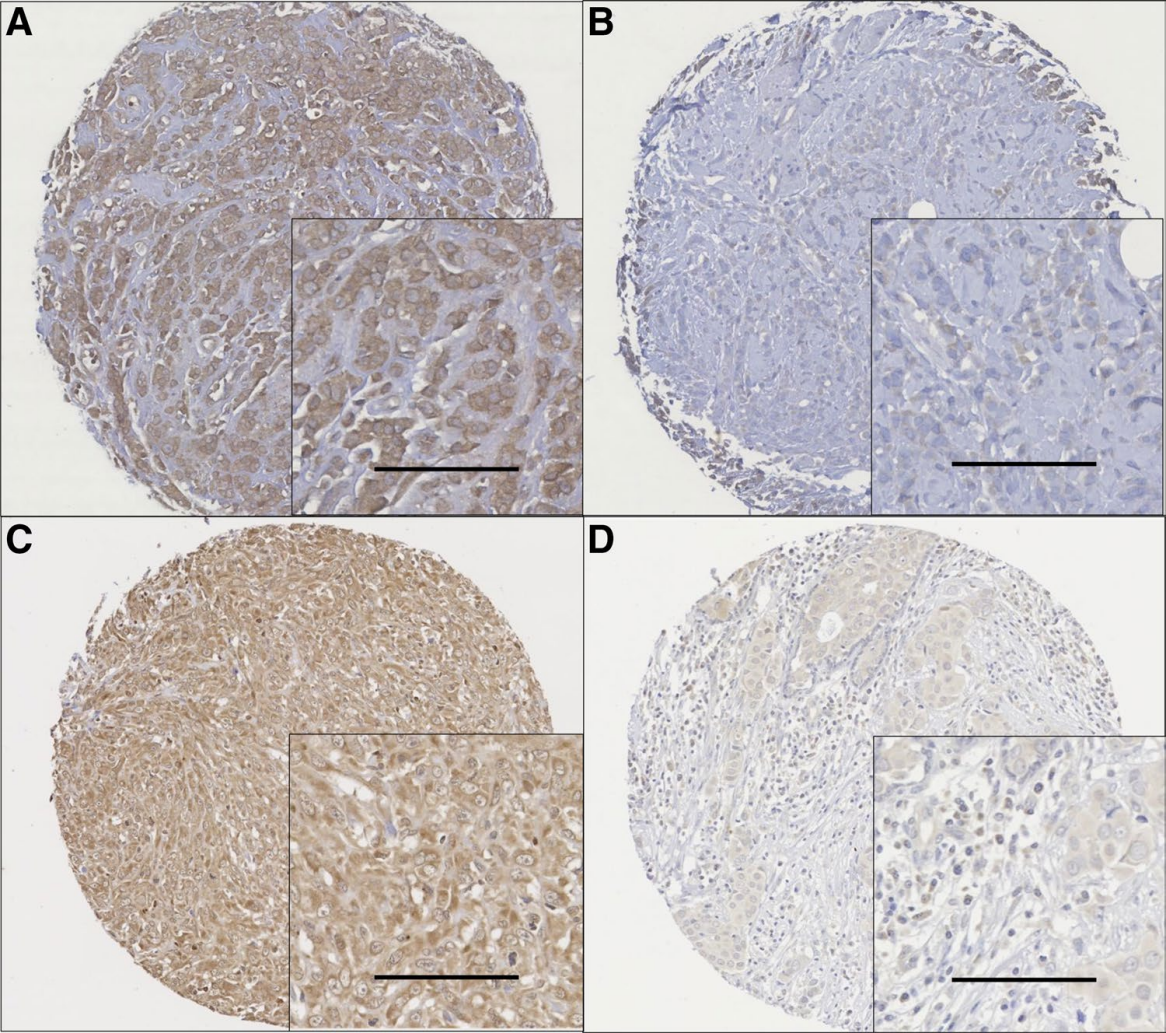
Representative photomicrographs at 10× magnification with 20× magnification *inset* panel and *scale bar* representing 100 μm. **a** Caspase-3 high expression; **b** caspase-3 low expression; **c** caspase-8 high expression; **d** caspase-8 low expression

Representative staining patterns are shown in Fig. 2. Caspase-3 and -8 expression was mainly granular/diffuse cytoplasmic staining with some heterogeneity between adjacent tumour cells, varying from weak to intense staining. Nuclear staining was observed in a small number of cases and some inflammatory cells were observed to express caspase-3 and -8. A few TMA cores were not assessed due to insufficient tumour or the core being missing, a total number of 1421 cases for caspase-3, and 1402 cases for caspase-8 were assessed. Caspase-3 had a median H-score of 80 ± 74 and ranged from 0 to 260; caspase-8 had a median H-score of 185 ± 43 and ranged from 0 to 275. The X-tile cut point for caspase-3 was 128, with 556 (39.8%) cases having high expression and 855 (60.2%) cases having low expression. The X-tile cut point for caspase-8 was 183, with 736 (52.5%) cases having high expression and 666 (47.5%) cases having low expression.

Spearman’s rank correlation was used to assess the correlation between caspase-3/-8, calpains, and calpastatin protein expression (Table 2). As immunohistochemical expression data for calpain-1, -2, -9 and calpastatin, were available, and as there are links in the literature between both systems it was decided to explore for potential correlations between calpain system protein expression and caspase-3/-8 protein expression. It would be interesting to further investigate the correlations between caspase-3/-8 and other calpain family members once such data becomes available. Correlations were found between the expression of caspase-3 and calpain-1 (r = 0.062, *P* = 0.047) and between caspase-8 and calpastatin (r = 0.086, *P* = 0.008); although statistically significant, these correlations are of marginal biological relevance, as reflected by the low r values. In breast cancer subgroup specific settings, significant correlations were found between caspase-3 and calpain-2 expression in the basal-like subgroup (r = 0.143, *P* = 0.045); caspase-3 and calpain-2 expression in the triple-negative subgroup (r = 0.194, *P* = 0.01); caspase-8 and calpain-9 (r = 0.25, *P* = 0.019), caspase-8 and calpastatin (r = 0.22, *P* = 0.009) in the HER2+ subgroup (Table 2) but, as before, are of marginal biological relevance due to the low r values.

**Table 2:** Correlation between protein expressions in total and different subgroups

	Total		HER2+		Basal-like		Triple-negative	
	R-value	Sig.	R-value	Sig.	R-value	Sig.	R-value	Sig.
Caspase-3								
Calpain-1	0.062	**0.047**	0.126	0.122	0.047	0.506	0.076	0.308
Calpain-2	0.062	0.051	−0.057	0.49	0.143	**0.045**	0.194	**0.01**
Calpain-9	0.031	0.443	0.088	0.408	−0.067	0.467	−0.056	0.557
Calpastatin	−0.053	0.099	−0.028	0.745	0.058	0.418	0.009	0.908
Caspase-8								
Calpain-1	−0.053	0.092	−0.067	0.402	0.05	0.485	−0.003	0.966
Calpain-2	0.017	0.606	0.062	0.461	0.088	0.228	0.013	0.868
Calpain-9	0.047	0.251	0.25	**0.019**	−0.005	0.957	−0.089	0.358
Calpastatin	0.086	**0.008**	0.22	**0.009**	0.047	0.515	−0.009	0.904

Significant *P*-values are indicated by bold font

### Relationship with clinicopathologic criteria

The expression of both caspase-3 and -8 was assessed for associations with clinicopathologic variables. High caspase-3 expression was significantly associated with HER2 positivity (χ^2^=6.624; df=1; *P* = 0.01). No significant association was found between caspase-8 expression and clinicopathologic variables (Table 3). High caspase-3 expression was significantly associated with the occurrence of death due to breast cancer (χ^2^=6.711; df=1; *P* = 0.01), recurrence (χ^2^=8.111; df=1; *P* = 0.004) and distant metastasis (χ2 = 5.724; df=1; *P* = 0.017) (Table 3).

**Table 3:** Correlations between caspase-3/-8 protein expressions and clinicopathologic variables

Variables	Caspase-3 (N = 1421)			Caspase-8 (N = 1402)		
	Low	High	*P*-value	Low	High	*P*-value
Age (years)						
≤40	91 (6.4%)	45 (3.2%)	0.09	66 (4.7%)	65 (4.6%)	0.494
>40	763 (53.7%)	521 (36.7%)		600 (42.8%)	670 (47.8%)	
Tumour size (mm)						
≤20	512 (36.2%)	341 (24.1%)	0.943	410 (29.4%)	432 (30.9%)	0.241
>20	339 (23.9%)	224 (15.8%)		252 (18.1%)	302 (21.6%)	
Tumour stage						
I	517 (36.5%)	353 (24.9%)	0.227	419 (30.0%)	453 (32.4%)	0.578
II	267 (18.9%)	156 (11.0%)		191 (13.7%)	212 (15.2%)	
III	68 (4.8%)	55 (3.9%)		52 (3.7%)	69 (4.9%)	
Tumour grade						
I	148 (10.5%)	97 (6.9%)	0.979	122 (8.7%)	114 (8.2%)	0.219
II	274 (19.4%)	180 (12.7%)		219 (15.7%)	234 (16.8%)	
III	429 (30.3%)	288 (20.3%)		321 (23.0%)	386 (27.7%)	
NPI						
≤3.4	251 (17.7%)	175 (12.4%)	0.416	203 (14.6%)	215 (15.4%)	0.516
3.41–5.4	452 (31.9%)	280 (19.8%)		348 (24.9%)	378 (27.1%)	
>5.4	148 (10.5%)	109 (7.7%)		111 (8.0%)	140 (10.0%)	
LVI						
Positive	238 (20.1%)	162 (13.7%)	0.456	117 (15.3%)	207 (17.9%)	0.747
Negative	484 (40.9%)	300 (25.3%)		349 (30.1%)	425 (36.7%)	
Operation type						
Mastectomy	493 (35.0%)	325 (23.1%)	0.99	378 (27.2%)	429 (30.9%)	0.511
WLE	356 (25.3%)	235 (16.7%)		283 (20.4%)	299 (21.5%)	
ER status						
Positive	614 (44.5%)	394 (28.6%)	0.526	463 (34.1%)	532 (39.1%)	0.439
Negative	219 (15.9%)	152 (11.0%)		178 (13.1%)	186 (13.7%)	
PR status						
Positive	467 (35.1%)	293 (22.0%)	0.35	349 (26.4%)	400 (30.3%)	0.469
Negative	337 (25.3%)	235 (17.6%)		278 (21.0%)	294 (22.3%)	
HER2 status						
Positive	100 (7.2%)	94 (6.8%)	**0.01**	84 (6.2%)	110 (8.1%)	0.201
Negative	729 (52.7%)	460 (33.3%)		565 (41.4%)	606 (44.4%)	
Basal status						
Positive	164 (12.4%)	110 (8.3%)	0.938	140 (10.7%)	128 (9.8%)	0.098
Negative	630 (47.7%)	418 (31.6%)		482 (37.0%)	553 (42.4%)	
Triple-negative status						
Positive	154 (11.2%)	96 (7.0%)	0.604	124 (9.2%)	122 (9.0%)	0.27
Negative	673 (48.9%)	452 (32.9%)		516 (38.1%)	593 (43.8%)	
Breast cancer-specific survival						
Alive	577 (41.7%)	342 (24.7%)	**0.01**	444 (32.6%)	464 (34.0%)	0.157
Dead	259 (18.7%)	207 (14.9%)		204 (15.0%)	251 (18.4%)	
Recurrence						
Present	318 (23.0%)	255 (18.5%)	**0.004**	263 (19.3%)	305 (22.4%)	0.496
Not present	510 (36.9%)	298 (21.6%)		382 (28.1%)	411 (30.2%)	
Distant metastasis						
Present	250 (17.7%)	201 (14.2%)	**0.017**	204 (14.7%)	240 (17.2%)	0.431
Not present	597 (42.3%)	364 (25.8%)		457 (32.8%)	491 (35.3%)	

*NPI* Nottingham Prognostic Index, *LVI* lymphovascular invasion (determined using IHC), *WLE* wide local excision, *ER* oestrogen receptor, *PR* progesterone receptor, *HER2* human epidermal growth factor receptor 2. Correlations between caspase-3/-8 protein expression and clinicopathologic variables was assessed using Pearson’s Chi square test of association (χ²) or Fisher’s exact test if in a 2 × 2 tables and cell count was <5. Significant *P*-values are indicated by bold font

### Relationship with clinical outcome

**Fig. 3:**
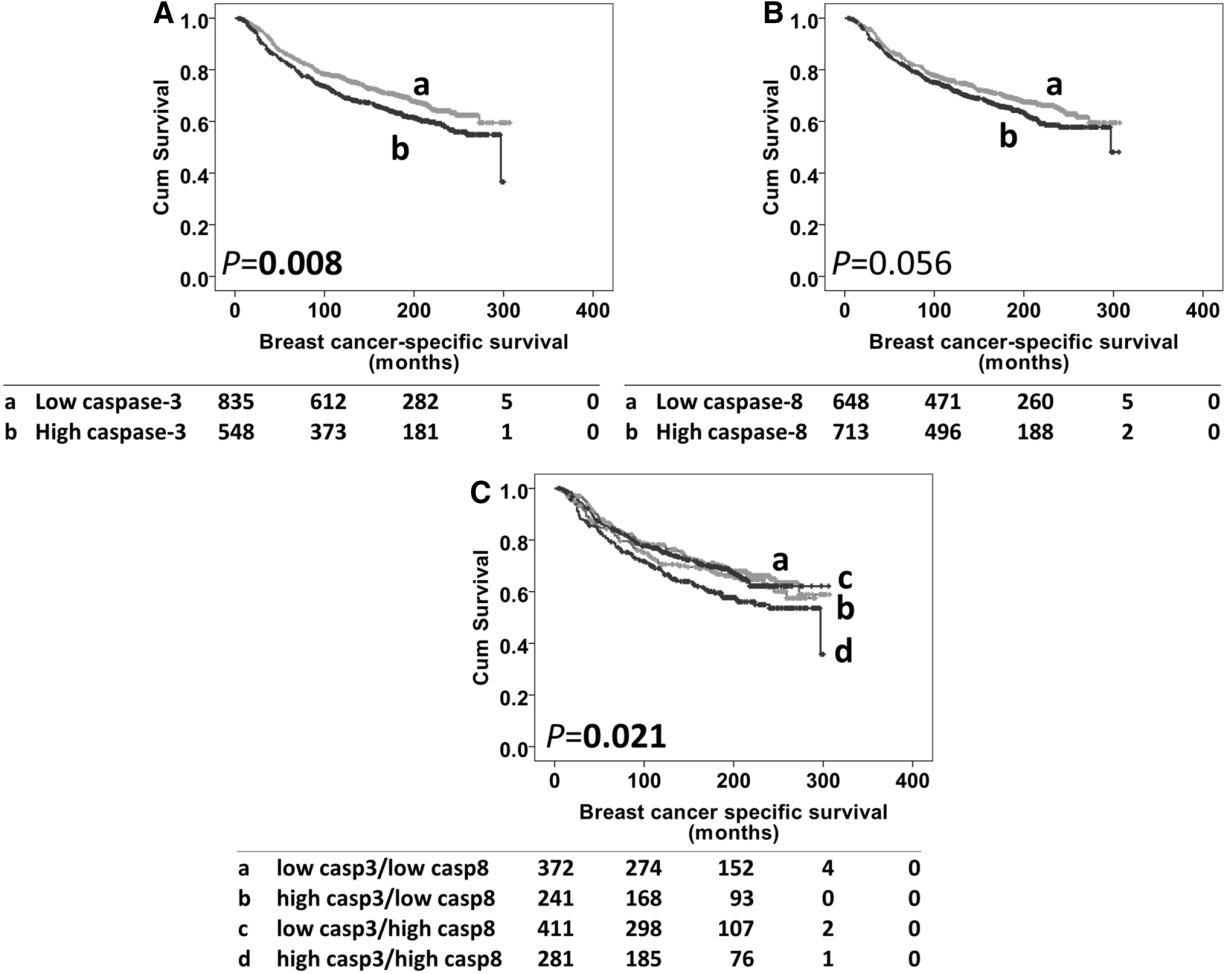
Kaplan–Meier survival curve analysis showing caspase-3 (*panel A*), caspase-8 (*panel B*) and combinatorial caspase-3 and -8 expression (*panel C*), related breast cancer-specific survival; significance was determined using the log rank test. Numbers below the graph show patients at risk at the specified months. *Panel A* low caspase-3 (**a**) and high caspase-3 (**b**) expression. *Panel B* low caspase-8 (**a**) and high caspase-8 (**b**) expression. *Panel C* low caspase-3/-8 (**a**), high caspase-3/low caspase-8 (**b**), low caspase-3/high caspase-8 (**c**), high caspase-3/high caspase-8 (**d**)

High caspase-3, but not caspase-8, expression was significantly associated with adverse disease-specific survival (*P* = 0.008 and 0.056 respectively) (Fig. 3a, b). In multivariate analysis, including potentially confounding factors of age, tumour size, stage, grade, NPI, lymphovascular invasion, ER, PR and HER2 status (with individual Kaplan–Meier statistics of *P* < 0.05 for all variables), caspase-3 expression remained significant for breast cancer-specific survival (hazard ratio (HR) 1.347, 95% confidence interval (CI) 1.086–1.670; *P* = 0.007) (Table 4). The caspase-3 and -8 expression was combined to assess their relationship with survival. The combination of high caspase-3 and -8 expression was significantly associated with adverse breast cancer-specific survival in the total patient cohort (*P* = 0.021) (Fig. 3c); significance was not, however, retained in multivariate analysis using the previous confounding variables (HR 1.069, 95% CI 0.967–1.182; *P* = 0.194).

**Table 4:** Cox proportional hazards analysis for breast cancer-specific survival

	Sig.	Exp(B)	95% CI for Exp(B)	
			Lower	Upper
Age	0.939	1.013	0.724	1.418
Tumour size	**0.003**	1.539	1.163	2.037
Tumour stage	<**0.001**	1.779	1.375	2.302
Tumour grade	**0.001**	1.595	1.197	2.125
Nottingham prognosis index	0.782	0.944	0.629	1.418
Lymphovascular invasion	<**0.001**	1.595	1.276	1.993
ER status	**0.009**	1.528	1.109	2.104
PR status	**0.001**	0.624	0.469	0.831
HER2 status	**0.035**	1.362	1.022	1.816
Caspase-3	**0.007**	1.347	1.086	1.670

*Exp (B)* hazard ratio, *95% CI* 95% confidence interval, *ER* oestrogen receptor, *PR* progesterone receptor, *HER2* human epidermal growth factor receptor 2. Significant *P*-values are indicated by bold font

**Fig. 4:**
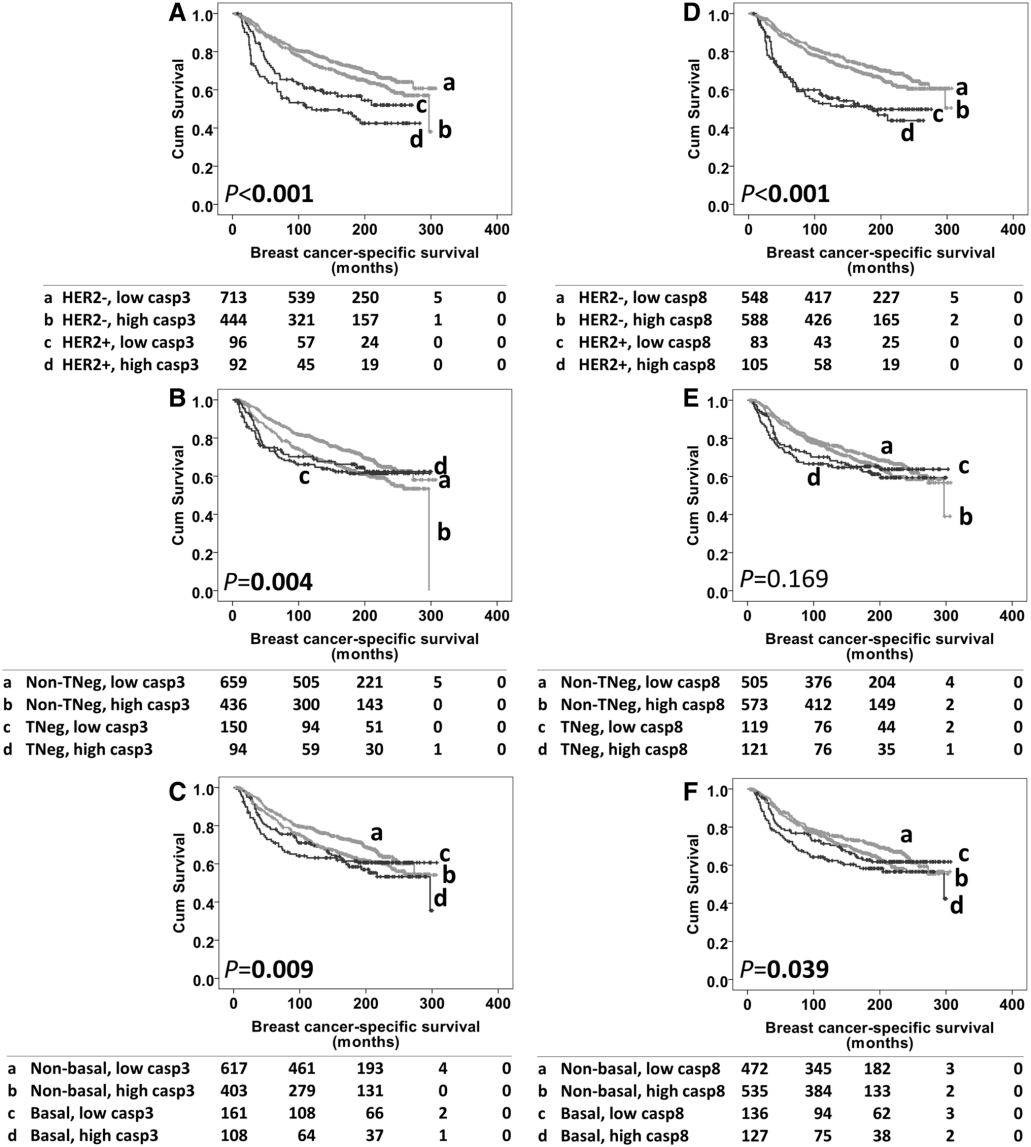
Kaplan–Meier survival curves for breast cancer specific-survival based upon caspase-3/caspase-8 expression, showing as different subgroups. *Panel A* caspase-3 expression with HER2 negative and HER2 positive diseases. *Panel B* caspase-3 expression with non-triple negative and triple-negative diseases. *Panel C* caspase-3 expression with non-basal like diseases and basal-like diseases. *Panel D* caspase-8 expression with HER2 negative and HER2 positive diseases. *Panel E* caspase-8 expression with non-triple negative and triple-negative diseases. *Panel F* caspase-8 expression with non-basal like diseases and basal-like diseases

**Fig. 5:**
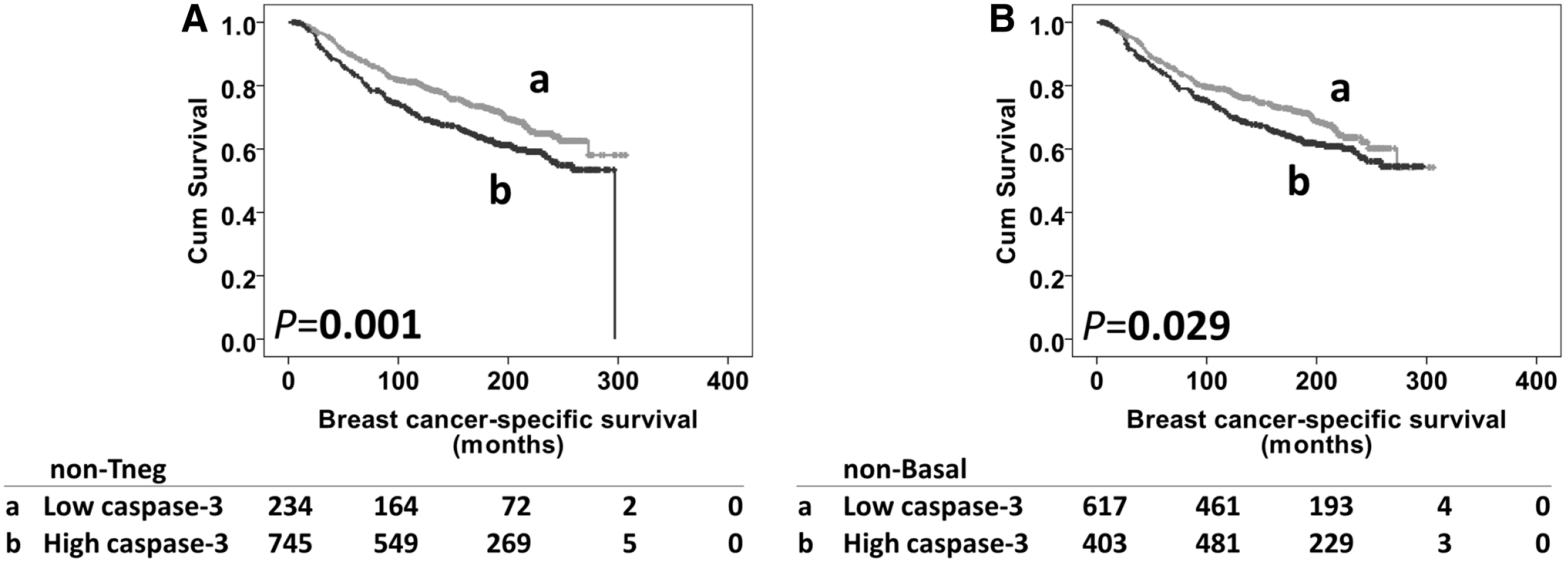
Kaplan–Meier survival curves for breast cancer-specific survival based upon caspase-3 expression in receptor positive subgroup (*panel A*) and non-basal like subgroup (*panel B*). Significance was determined using the log rank test. Numbers below the graph show patients at risk at the specified months. *Panel A, B* high caspase-3 expression (**a**) and low caspase-3 expression (**b**)

Caspase-3 and -8 expressions were examined for associations with breast cancer-specific survival in different breast cancer subgroups. When analysed in the total patient cohort, caspase-3 expression showed interest in HER2 positive, triple-negative and basal-like diseases (*P* < 0.001, *P* = 0.004 or *P* = 0.009, respectively, Fig. 4a–c); whilst caspase-8 expression showed interest in HER2+ and basal-like disease (*P* < 0.001 or *P* = 0.039, respectively, Fig. 4d, f). This was further investigated in the individual subgroups (HER2 positive, triple-negative and basal-like). Neither caspase-3 nor caspase-8 protein expression was significantly associated with breast cancer-specific survival in any of the groups (data not shown). In comparison, caspase-3 expression was associated with survival in receptor positive (ER, PR or HER2) and non-basal like subgroups (*P* = 0.001 and 0.029, Fig. 5a, b). In the receptor positive subgroup, significance was retained in multivariate analysis (HR 1.465, 95% CI 1.150–1.867; *P* = 0.002), including confounding factors of age, tumour size, stage, grade, NPI, lymphovascular invasion, ER, PR and HER2 status (with individual Kaplan–Meier statistics of *P* < 0.05 for all variables). The significance was also retained in non-basal like patients for multivariate analysis (HR 1.385, 95% CI 1.079–1.777; *P* = 0.01), including the same confounding factors as in receptor positive subgroup (with individual Kaplan–Meier statistics of *P* < 0.05 for all variables).

### Combinational biomarker analysis

The relationship between caspase and calpain expression with breast cancer-specific survival was explored. The expression of proteins was grouped (e.g. high caspase/high calpain, low caspase/low calpain, high caspase/low calpain, and low caspase/high calpain). Patients with high caspase-3/high calpain-1 expression, high caspase-3/high calpain-2 expression, high caspase-3/low calpastatin expression, or high caspase-8/low calpain-1 expression had significantly worse breast cancer-specific survival (*P* = 0.005, 0.049, 0.02, and 0.02 respectively) (Table 5). The significance of caspase-3/calpastatin for breast cancer-specific survival was retained in multivariate analysis (HR 1.241, 95% CI 1.053–1.462; *P* = 0.01), when considering previous confounding factors. No other significant association was found between any combinational protein expressions and breast cancer-specific survival in the total patient cohort (data not shown). In subgroup analyses, a significant association was found between caspase-3/calpain-1 and breast cancer-specific survival in the basal-like subgroup (*P* = 0.034), the same pattern shown in the total group (high caspase-3/high calpain-1) that was associated with poor prognosis. However, significance was not retained from multivariate analysis, using previous confounding factors.

**Table 5:** Kaplan Meier survival analyses for breast cancer-specific survival based upon combinatorial protein expression

	Calpain-1		Calpain-2		Calpain-9		Calpastatin	
	*P*-value	Patients (N)	*P*-value	Patients (N)	*P*-value	Patients (N)	*P*-value	Patients (N)
Total								
Caspase-3	**0.005**	994	**0.049**	998	0.139	589	**0.02**	951
Caspase-8	**0.02**	971	0.056	943	0.072	570	0.085	930
HER2+								
Caspase-3	0.575	145	0.855	141	0.248	89	0.244	135
Caspase-8	0.382	142	0.706	138	0.779	86	0.49	133
Basal-like								
Caspase-3	**0.034**	197	0.059	193	0.253	118	0.228	194
Caspase-8	0.25	190	0.054	185	0.308	114	0.322	187
TNeg								
Caspase-3	0.109	175	0.292	172	0.205	108	0.654	175
Caspase-8	0.579	166	0.235	163	0.071	103	0.291	166

Kaplan–Meier survival analyses for breast cancer-specific survival based upon combined protein expression in the total patient cohort and different breast tumour phenotypes. Significance was determined using the log-rank test. Significant *P* values are indicated by bold font. *HER2* human epidermal growth factor receptor 2, *TNeg* triple-negative

## Discussion

High caspase-3 expression is significantly associated with adverse disease-specific survival (*P* = 0.008); and remains significant in multivariate analysis when considering potential confounding factors (*P* = 0.007). Caspase-8 was not associated with breast cancer-specific survival. Several studies have suggested that caspase-3 overexpression and enhanced apoptotic activity, is observed in breast carcinomas compared with controls; however, none have shown significant associations between protein expression and patient prognosis [21, 35]. One study demonstrated that 75% (35 out of 46) of breast tumours lacked caspase-3 transcript and protein expression, and the remaining samples had substantially decreased expression [11]. This finding was in contrast with previous reports by Nakopoulou et al., in which high caspase-3 protein expression was present in 75.2% (103 out of 137) of invasive breast tumour samples, in comparison with non-neoplastic breast tissues [36]. Results from the current study suggest that high caspase-3 expression is associated with adverse breast cancer-specific survival in breast cancer patients.

No associations were observed between caspase-8 and clinicopathological criteria. High caspase-3 expression was significantly associated with HER2 positive tumours (*P* = 0.01). Associations between HER2 positive tumours and caspase-3 expression have not been reported previously; however, activation of HER2 can trigger downstream signalling via the phosphatidylinositol kinase 3 (PI3K/AKT) pathways, and AKT signalling has been shown to reduce the activity of caspase-9, the downstream effector of caspase-3 [37, 38]. Expression of phosphorylated AKT and phosphorylated caspase-9 are significantly correlated in gastric and colorectal cancer [39]. As discussed before, caspase-3 is the downstream effector of caspase-9; it may be possible that caspase-3 interacts with other substrates and potentially regulates apoptosis through the HER2 signalling pathway.

The prognostic significance of caspase-3 expression in different breast cancer phenotypes was also examined. The Kaplan–Meier subgroup analyses demonstrated a significant association in receptor positive (ER, PR or HER2) and non-basal like subgroups, and remained significant in multivariate analysis. In the case of caspase-8, no significant association was observed in any individual subgroup. The results further emphasised the prognostic importance of caspase-3 in receptor positive and non-basal like patients.

Calpain plays an important role in tumorigenesis, and there is a close link between calpain and caspase protein families. We have previously shown that high calpain-2 expression is associated with worse prognosis in patients with basal-like or triple-negative diseases [23] and that calpain-1 expression is associated with trastuzumab response in HER2+ breast cancer patients [27, 33]. Calpain and caspase protein expression was combined to assess associations with breast cancer-specific survival. High caspase-3/high calpain-1, high caspase-3/high calpain-2 and high caspase-3/low calpastatin expression significantly associated with adverse breast cancer-specific survival in the total patient cohort; and combinational caspase-3/calpain-1 has important prognostic value in basal-like patients. Only caspase-3/calpastatin expression was identified as an independent factor for breast cancer-specific survival from multivariate analysis. It is important to note that this study focuses on the expression levels of functionally significant proteases, without assessing their activity. It would be interesting to investigate the relationship between patient prognosis and active caspases and calpains as part of any future work. Antibodies are available for caspase-3 and -8 and different methodologies are used to detect their activities; however no published studies have investigated their expression in breast cancer [40, 41]. Antibodies have been described that are able to determine calpain activity through the detection of specific calpain-cleaved products; however these antibodies have not been optimised for use in human tumour tissue [42, 43].

The current study demonstrates that high caspase-3 expression is significantly associated with adverse breast cancer-specific survival, and remains so in multivariate analysis including potential confounding factors. Caspase-8 expression is not associated with breast cancer-specific survival. Further subgroup analyses demonstrate that caspase-3 and -8 expression are important in distinct breast cancer phenotypes. Caspase-3 and -8 expression was combined with calpain expression to provide important information on evaluating patient outcome in basal-like phenotype disease. Further validation is warranted to confirm if determining caspase-3 with/without calpain expression would be of clinical benefit.

## References

[1] Hengartner MO (2000). The biochemistry of apoptosis. Nature.

[2] Pop C, Salvesen GS (2009). Human caspases: activation, specificity, and regulation. J Biol Chem.

[3] Shi Y (2002). Mechanisms of caspase activation and inhibition during apoptosis. Mol Cell.

[4] Estrov Z, Thall PF, Talpaz M, Estey EH, Kantarjian HM, Andreeff M, Harris D, Van Q, Walterscheid M, Kornblau SM (1998). Caspase-2 and caspase-3 protein levels as predictors of survival in acute myelogenous leukemia. Blood.

[5] Winter RN, Kramer A, Borkowski A, Kyprianou N (2001). Loss of caspase-1 and caspase-3 protein expression in human prostate cancer. Cancer Res.

[6] Teitz T, Wei T, Valentine MB, Vanin EF, Grenet J, Valentine VA, Behm FG, Look AT, Lahti JM, Kidd VJ (2000). Caspase 8 is deleted or silenced preferentially in childhood neuroblastomas with amplification of MYCN. Nat Med.

[7] Shivapurkar N, Toyooka S, Eby MT, Huang CX, Sathyanarayana UG, Cunningham HT, Reddy JL, Brambilla E, Takahashi T, Minna JD (2002). Differential inactivation of caspase-8 in lung cancers. Cancer Biol Ther.

[8] Umar M, Upadhyay R, Kumar S, Ghoshal UC, Mittal B (2011). CASP8–652 6 N del and CASP8 IVS12-19G> A gene polymorphisms and susceptibility/prognosis of ESCC: a case control study in northern Indian population. J Surg Oncol.

[9] Yin J, Tang W, Shao A, Wang L, Wang X, Ding G, Liu C, Chen Y, Chen S, Gu H (2014). Caspase8 rs1035142 G> T polymorphism was associated with an increased risk of esophageal cancer in a Chinese population. Mol Biol Rep.

[10] Yang X-H, Sladek TL, Liu X, Butler BR, Froelich CJ, Thor AD (2001). Reconstitution of caspase 3 sensitizes MCF-7 breast cancer cells to doxorubicin-and etoposide-induced apoptosis. Cancer Res.

[11] Devarajan E, Sahin AA, Chen JS, Krishnamurthy RR, Aggarwal N, Brun A-M, Sapino A, Zhang F, Sharma D, Yang X-H (2002). Down-regulation of caspase 3 in breast cancer: a possible mechanism for chemoresistance. Oncogene.

[12] Blanc C, Deveraux QL, Krajewski S, Jänicke RU, Porter AG, Reed JC, Jaggi R, Marti A (2000). Caspase-3 is essential for procaspase-9 processing and cisplatin-induced apoptosis of MCF-7 breast cancer cells. Cancer Res.

[13] Goll DE, Thompson VF, Li H, Wei W, CONG J (2003). The calpain system. Physiol Rev.

[14] Wendt A, Thompson VF, Goll DE (2004). Interaction of calpastatin with calpain: a review. Biol Chem.

[15] Atencio IA, Ramachandra M, Shabram P, Demers GW (2000). Calpain inhibitor 1 activates p53-dependent apoptosis in tumor cell lines. Cell Growth Differ.

[16] Gafni J, Cong X, Chen SF, Gibson BW, Ellerby LM (2009). Calpain-1 cleaves and activates caspase-7. J Biol Chem.

[17] Tan Y, Dourdin N, Wu C, De Veyra T, Elce JS, Greer PA (2006). Ubiquitous calpains promote caspase-12 and JNK activation during endoplasmic reticulum stress-induced apoptosis. J Biol Chem.

[18] Chua BT, Guo K, Li P (2000). Direct cleavage by the calcium-activated protease calpain can lead to inactivation of caspases. J Biol Chem.

[19] Yoo Jy, Kim CH, Song SH, Shim BY, Jeong YJ, Ahn MI, Kim S, Cho DG, Jo MS, Cho KD, Cho HJ, Kang SJ, Kim HK (2004). Expression of Caspase-3 and c-myc in Non-Small Cell Lung Cancer. Cancer Res Treat.

[20] Huang H, Zhang XF, Zhou HJ, Xue YH, Dong QZ, Ye QH, Qin LX (2010). Expression and prognostic significance of osteopontin and caspase-3 in hepatocellular carcinoma patients after curative resection. Cancer Sci.

[21] Blazquez S, Sirvent JJ, Olona M, Aguilar C, Pelegri A, Garcia JF, Palacios J (2006). Caspase-3 and caspase-6 in ductal breast carcinoma: a descriptive study. Histol Histopathol.

[22] Storr SJ, Mohammed RA, Woolston CM, Green AR, Parr T, Spiteri I, Caldas C, Ball GR, Ellis IO, Martin SG (2011). Calpastatin is associated with lymphovascular invasion in breast cancer. The Breast.

[23] Storr SJ, Lee KW, Woolston CM, Safuan S, Green AR, Macmillan RD, Benhasouna A, Parr T, Ellis IO, Martin SG (2012). Calpain system protein expression in basal-like and triple-negative invasive breast cancer. Ann Oncol.

[24] Storr SJ, Safuan S, Woolston CM, Abdel-Fatah T, Deen S, Chan SY, Martin SG (2012). Calpain-2 expression is associated with response to platinum based chemotherapy, progression-free and overall survival in ovarian cancer. J Cell Mol Med.

[25] Storr SJ, Zaitoun AM, Arora A, Durrant LG, Lobo DN, Madhusudan S, Martin SG (2012). Calpain system protein expression in carcinomas of the pancreas, bile duct and ampulla. BMC Cancer.

[26] Storr SJ, Pu X, Davis J, Lobo D, Reece-Smith AM, Parsons SL, Madhusudan S, Martin SG (2013). Expression of the calpain system is associated with poor clinical outcome in gastro-oesophageal adenocarcinomas. J Gastroenterol.

[27] Pu X, Storr SJ, Ahmad NS, Chan SY, Moseley PM, Televantou D, Cresti N, Boddy A, Ellis IO, Martin SG (2016). Calpain-1 is associated with adverse relapse free survival in breast cancer: a confirmatory study. Histopathology.

[28] Abdel-Fatah TM, Powe DG, Agboola J, Adamowicz-Brice M, Blamey RW, Lopez-Garcia MA, Green AR, Reis-Filho JS, Ellis IO (2010). The biological, clinical and prognostic implications of p53 transcriptional pathways in breast cancers. J Pathol.

[29] Rakha EA, El-Rehim DA, Paish C, Green AR, Lee AH, Robertson JF, Blamey RW, Macmillan D, Ellis IO (2006). Basal phenotype identifies a poor prognostic subgroup of breast cancer of clinical importance. Eur J Cancer.

[30] McShane LM, Altman DG, Sauerbrei W, Taube SE, Gion M, Clark GM (2005). REporting recommendations for tumour MARKer prognostic studies (REMARK). Br J Cancer.

[31] Davis J, Martin SG, Patel PM, Green AR, Rakha EA, Ellis IO, Storr SJ (2014). Low calpain-9 is associated with adverse disease-specific survival following endocrine therapy in breast cancer. BMC Cancer.

[32] Abd El-Rehim DM, Ball G, Pinder SE, Rakha E, Paish C, Robertson JFR, Macmillan D, Blamey RW, Ellis IO (2005). High-throughput protein expression analysis using tissue microarray technology of a large well-characterised series identifies biologically distinct classes of breast cancer confirming recent cDNA expression analyses. Int J Cancer.

[33] Storr SJ, Woolston CM, Barros FFT, Green AR, Shehata M, Chan SY, Ellis IO, Martin SG (2011). Calpain-1 expression is associated with relapse-free survival in breast cancer patients treated with trastuzumab following adjuvant chemotherapy. Int J Cancer.

[34] Camp RL, Dolled-Filhart M, Rimm DL (2004). X-tile a new bio-informatics tool for biomarker assessment and outcome-based cut-point optimization. Clin Cancer Res.

[35] Vakkala M, Pääkkö P, Soini Y (1999). Expression of caspases 3, 6 and 8 is increased in parallel with apoptosis and histological aggressiveness of the breast lesion. Br J Cancer.

[36] Nakopoulou L, Alexandrou P, Stefanaki K, Panayotopoulou E, Lazaris AC, Davaris PS (2001). Immunohistochemical expression of caspase-3 as an adverse indicator of the clinical outcome in human breast cancer. Pathobiology.

[37] Rimawi MF, Schiff R, Osborne CK (2015). Targeting HER2 for the treatment of breast cancer. Annu Rev Med.

[38] Cardone MH, Roy N, Stennicke HR, Salvesen GS, Franke TF, Stanbridge E, Frisch S, Reed JC (1998). Regulation of cell death protease caspase-9 by phosphorylation. Science.

[39] Sangawa A, Shintani M, Yamao N, Kamoshida S (2014). Phosphorylation status of Akt and caspase-9 in gastric and colorectal carcinomas. Int J Clin Exp Pathol.

[40] Zhang X, Chen W, De Paiva CS, Corrales RM, Volpe EA, McClellan AJ, Farley WJ, Li D-Q, Pflugfelder SC (2011). Interferon-γ Exacerbates Dry Eye–Induced Apoptosis in Conjunctiva through Dual Apoptotic Pathways. Invest Ophthalmol Vis Sci.

[41] Berg C, Engels I, Rothbart A, Lauber K, Renz A, Schlosser S, Schulze-Osthoff K, Wesselborg S (2001). Human mature red blood cells express caspase-3 and caspase-8, but are devoid of mitochondrial regulators of apoptosis. Cell Death Differ.

[42] Dutta S, Chiu YC, Probert AW, Wang KK (2002). Selective release of calpain produced αII-spectrin (α-fodrin) breakdown products by acute neuronal cell death. Biol Chem.

[43] Takamure M, Murata K-Y, Tamada Y, Azuma M, Ueno S (2005). Calpain-dependent α-fodrin cleavage at the sarcolemma in muscle diseases. Muscle Nerve.

